# E2F6 Associates with BRG1 in Transcriptional Regulation

**DOI:** 10.1371/journal.pone.0047967

**Published:** 2012-10-17

**Authors:** Janet Y. Leung, Joseph R. Nevins

**Affiliations:** 1 Duke Institute for Genome Sciences and Policy, Duke University Medical Center, Durham, North Carolina, United States of America; 2 Department of Molecular Genetics and Microbiology, Duke University Medical Center, Durham, North Carolina, United States of America; George Mason University, United States of America

## Abstract

The E2F6 protein functions as an Rb-independent repressor of gene transcription. We have previously provided evidence suggesting a role for E2F6 in repression of E2F-responsive genes at S phase. Here, we have identified BRG1, the ATPase subunit of the SWI/SNF chromatin-remodeling complex, as an E2F6 interacting protein. Immunoprecipitation experiments demonstrate that BRG1 binds specifically to E2F6 and E2F4 but not the activator E2Fs. E2F6 was also able to interact with BAF155, a BRG1-associated factor, in the SWI/SNF complex. Chromatin immunoprecipitation assays demonstrate the binding of BRG1 coincident with E2F6 on G1/S gene promoters during S phase. Collectively, our studies suggest that E2F6 may recruit BRG1 in transcriptional regulation of genes important for G1/S phase transition of the cell cycle.

## Introduction

Numerous studies have linked E2F activity to cell cycle control [1], [2], [3], [4]. These studies have delineated roles for individual E2Fs in regulating G1/S and G2/M phase transitions of the cell cycle through activation and repression of target genes [5], [6], [7]. The E2F family is comprised of eight distinct gene products (E2Fs 1–8) which can be divided into three subclasses based on shared functional properties and sequence homologies. E2F1, E2F2 and E2F3 function as activators of transcription and make up one subset. These activator E2Fs are tightly regulated with essentially no expression in quiescent cells and are dramatically induced as cells are stimulated to grow [8], [9], [10]. During mid-to-late G1 phase of cell cycle progression, many E2F-responsive promoters are bound by E2F1, E2F2 or E2F3 coincident with gene activation [6], [11]. E2F4 and E2F5 comprise the second subset of E2F family members. In contrast to the activating E2Fs, E2F4 and E2F5 lack an activation domain and function as repressors of transcription. In the cell cycle, E2F4 and E2F5 are mainly involved in the repression of growth promoting E2F-responsive genes [12], [13], [14]. Studies to elucidate the mechanism of E2F action revealed that these transcription factors modulate gene expression through the formation of coactivator or corepressor complexes that alter chromatin [6], [12], [13], [14], [15]. E2Fs1-3, for example, have been documented to recruit p300/CBP and PCAF/GCN5 histone acetyltransferases (HATs) to activate target promoters while E2F4 promoter occupancy has been linked to the Sin3B corepressor/HDAC complex [6], [16], [17].

E2F6, E2F7 and E2F8 comprise the third and most recently discovered group of E2Fs. These E2Fs are unique in that they lack the activation domain common to E2Fs1-3 and the RB-binding domain common to all other E2Fs [18], [19], [20], [21], [22], [23], [24]. Among this third group of E2Fs, the function of E2F6 has perhaps been the most investigated. Mouse knockout studies show E2f6-null mice are healthy and viable but display homeotic transformation of the axial skeleton suggesting a role for E2F6 in developmental patterning [25]. Additional work has suggested E2F6 recruits polycomb group proteins to function as a repressor of target genes during development [24]. We previously observed that E2F6 binds to a subset of E2F target genes during S phase, implicating a role for E2F6 in balancing the function of the activating E2Fs during G1-S phase transition of the cell cycle [5]. Moreover, we also observed in these studies that E2F4 was able to compensate for loss of E2F6 in E2F6-null mouse embryo fibroblast cells.

To gain a better understanding of the mechanisms of E2F6 action during cell cycle progression, we performed a yeast two hybrid screen to identify novel E2F6-interacting proteins. This led to the identification of BRG1, the ATPase subunit of the SWI/SNF chromatin-remodeling complex originally identified in screens for genes that regulate mating-type switching (SWI) and sucrose nonfermenting (SNF) phenotypes in yeasts [26], [27], [28], [29], [30]. Here, we show that BRG1 bound specifically to E2F6 and E2F4 but not the other E2Fs. E2F6 was also able to interact with BAF155, a BRG1-associated factor, in the SWI/SNF complex. Chromatin immunoprecipitation assays show that BRG1 associated with a G1/S promoter concurrent with E2F6 during S phase. Collectively, our studies suggest that E2F6 may function in transcriptional regulation of G1/S genes in the cell cycle by recruiting BRG1.

**Table 1:** E2F6-interacting proteins identified from a yeast two-hybrid screen.

Clone	Genbank Accession No.	E2F1	E2F2	E2F3	E2F4	E2F6
MIZ1	NM_003443	+	−	+	−	+
FAZF	NM_014383	+	−	+	−	+
DNMT1	NM_001379	+	−	−	−	+
BRG1*	NM_003072	−	−	−	−	+
NF-Yalpha	NM_021705	+	−	+	−	+
BRAF35	NM_006339	+	−	−	−	+
BRDT	NM_001726	+	−	−	−	+
ZFPM2	NM_012082	+	−	+	−	+
DNTTIP1	NM_052951	+	−	−	−	+
IN080B	NM_031288	+	−	−	−	+
EPC1	NM_025209	+	−	+	−	+
KLIP1	NM_024629	+	−	−	−	+
DP1	NM_026580	+	+	+	+	+
DP2	NM_001178138	+	+	+	+	+

Clones identified from the yeast two-hybrid screen were tested for an interaction with E2Fs1–4 and E2F6 in yeast. Full length human E2Fs 1–4 and E2F6 were cloned into pGBT9B as described in Materials and Methods and the resulting plasmid, along with DP1, was transformed into yeast to test for an interaction with the indicated proteins.

* Ectopic expression of E2F4 was able to immunoprecipitate ectopically expressed BRG1 in T98G cells even though an interaction was not observed in yeast.

## Materials and Methods

### Yeast two-hybrid screen

A GAL4-based yeast two-hybrid assay was performed according to recommendations from BD Biosciences with a few exceptions. In brief, full length E2F6 was cloned into the pGBT9B vector to create a GAL4 DNA-binding domain (DBD)-E2F6 fusion used as bait. E2F6-pGBT9 was introduced into the pJ69a yeast strain along with DP1-p2U and used to screen a BD Matchmaker Human Testis Library (BD Biosciences HY4035AH) pretransformed into the Y187 yeast strain. A mating efficiency of 9% was achieved and approximately 9×10^6^ clones were screened. Prey plasmids were rescued and transformed into the bacterial strain MH1066. The inserts recovered in the prey plasmids were then sequenced by the Duke DNA sequencing core facility.

**Figure 1:**
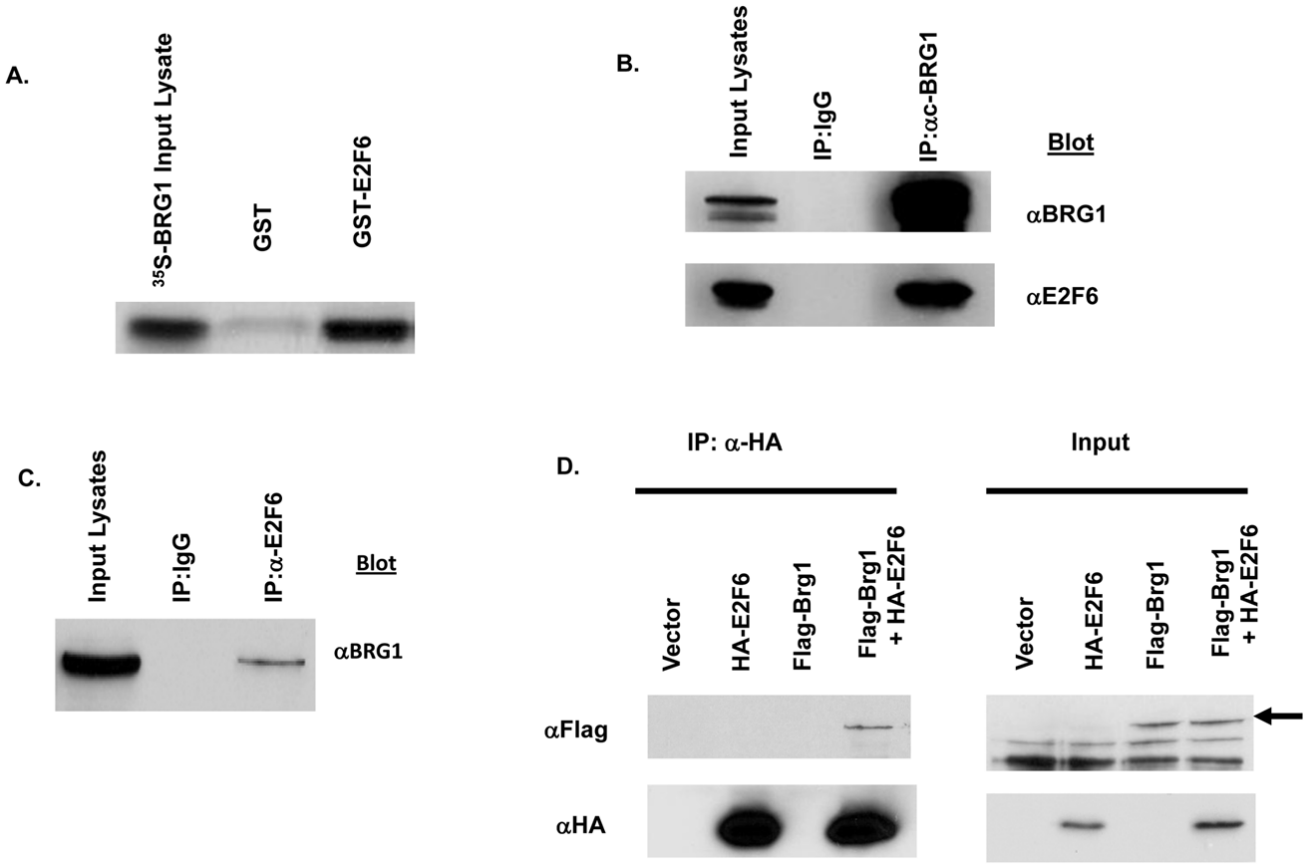
E2F6 interacts with BRG1. A) A GST-E2F6 recombinant protein directly interacts with *in vitro*-translated BRG1 proteins. GST-E2F6 recombinant proteins generated in *E-coli* were immobilized on glutathione S-transferase (GST)-Sepharose beads and incubated with S^35^-labeled *in vitro*-translated BRG1 proteins. E-coli expressed GST protein was used as a control. Beads were washed in TBS/Triton X-100 after four hours, resuspended in 2 X Laemelli's buffer and resolved on a 6% SDS PAGE gel. B) BRG1 coimmunoprecipitates with E2F6 proteins. Plasmid constructs overexpressing BRG1 and E2F6 were co-transfected into T98G cells. Immunoprecipitations were performed with a monoclonal antibody recognizing BRG1. Immunoprecipitates were resolved on SDS PAGE and assayed by Western blotting with polyclonal antibodies recognizing BRG1 or E2F6. C) Endogenous E2F6 and BRG1 proteins coimmunoprecipitates. Asynchronously growing 293 cells were collected in lysis buffer. Immunopreciptations were performed with a polyconal antibody recognizing E2F6. Immunoprecipitated proteins were resolved on SDS PAGE and assayed by Western blotting with a monoclonal antibody recognizing BRG1. D) HA-tagged E2F6 interacts with flag-tagged BRG1. Plasmid constructs overexpressing epitope-tagged versions of E2F6 and BRG1 were individually transfected or cotransfected into 293 cells. Immunoprecipitations were carried out with a polyclonal antibody recognizing the HA epitope tag on E2F6. Immunoprecipitates were resolved on SDS PAGE and Western blotted with the monoclonal M2 anti-flag antibody.

### In vitro binding assays

[^35^S] methionine-labeled BRG1 was synthesized with a coupled *in vitro* transcription and translation system as specified by the manufacturer (Promega). [^35^S] methionine-labeled BRG1 (20 ul) was added to GST or GST-E2F6 fused to glutathione S-transferase (GST)-Sepharose beads in 500 ul TBS/1% Triton X-100. The reaction was rotated at 4°C for 2 hours and GST beads were washed thoroughly with TBS/Triton X-100. Immunoprecipitated proteins were eluted by boiling in 2 X Laemelli's buffer and then separated by SDS-PAGE. Gels were stained, destained, soaked in Amplify (Amersham Biosciences), dried, and exposed to autoradiographic film.

**Figure 2:**
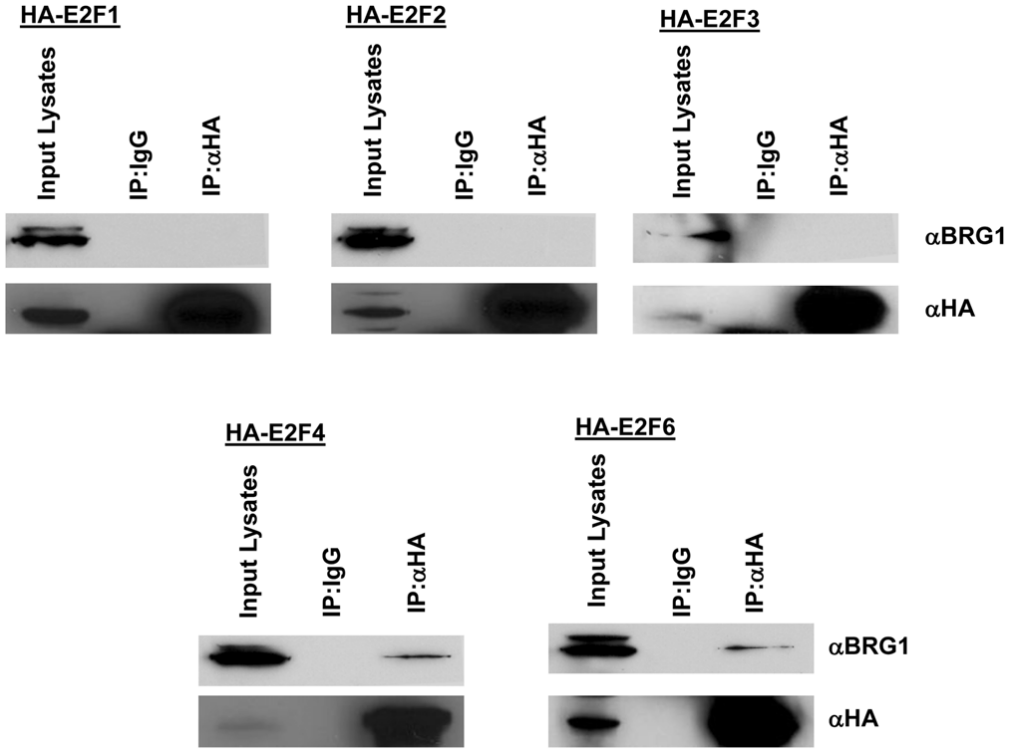
BRG1 interacts with the E2F4 and E2F6. BRG1 specifically interacts with E2F4 and E2F6 but not with the activator E2Fs. A plasmid construct expressing BRG1 was cotransfected into 293 cells with plasmid constructs expressing HA-tagged E2F1, E2F2, E2F3, E2F4 or E2F6. Immunoprecipitation experiments were carried out in lysis buffer with a polyclonal antibody recognizing the HA epitope tag. Immunoprecipitates were resolved on SDS PAGE and Western blotted with a BRG1 monoclonal antibody.

### Immunoprecipitations

T98G or 293 cells were transfected with the desired plasmids using Superfect Transfection Reagent (Qiagen). All expression plasmids were in a pcDNA3 vector backbone unless otherwise stated. Cell lysates were collected 36–48 hours post transfection in lysis buffer (50 mM Tris-HCl, pH 7.4, 150 mM NaCl, 1 mM EDTA, 1% Triton X-100) and incubated with the desired antibodies and Pro A or Pro G beads (Roche). Immunopreciptations to detect an interaction between endogenous E2F6 and BRG1 were performed on 293 cells with no transfections. Immunoprecipitates were eluted by boiling in 2 X Laemelli's buffer and then separated by SDS-PAGE. Western blots were carried out with the desired antibodies. E2F6, BRG1, DP1, BAF155 and HA proteins in immunopreciptiations and Western blots were carried out using antibodies from Santa Cruz (E2F6, sc-8175 & sc-8366; HA, sc-805 & sc-7392; BRG1, sc-17796 & sc-10768; DP1, sc-610 & sc-53642; BAF155, sc-32763). The antibody recognizing the Flag epitope tag was purchased from Sigma (F3165). The antibody recognizing BAF180 was purchased from Millipore (ABE70). The density of bands on Western blots were quantitated using the publicly available software, Image J.

**Figure 3:**
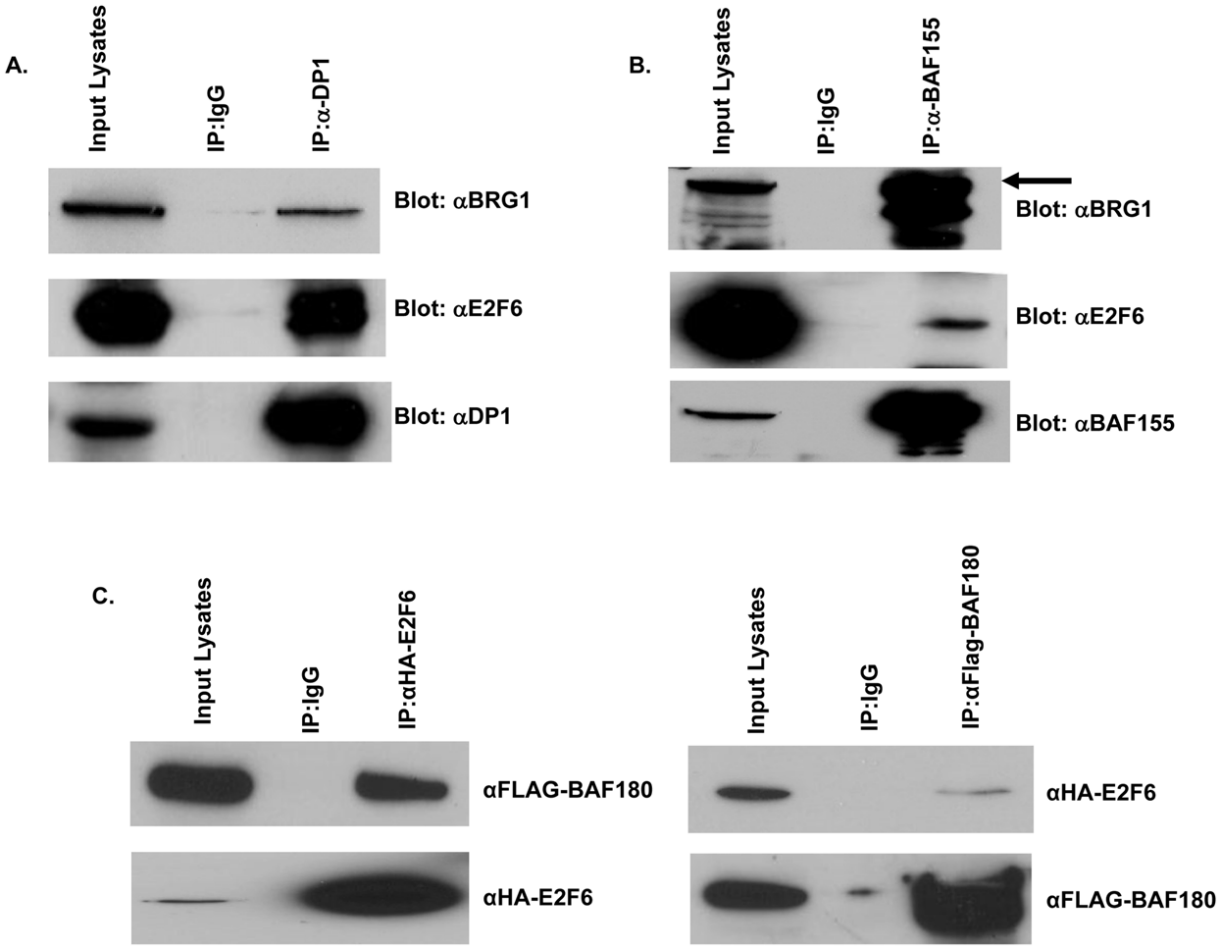
BRG1 and E2F6 functions in a complex with other proteins. A) DP1, previously shown to interact with E2F6, is also in a complex together with BRG 1. Plasmid constructs expressing DP1, E2F6, and BRG1 were contransfected into 293 cells. Immunoprecipitation experiments were carried out in lysis buffer with a monoclonal antibody recognizing DP1. Immunoprecipitates were resolved on SDS PAGE and Western blotted for E2F6, BRG1 and DP1. B) BAF155, previously shown to interact with BRG1, is also in a complex together with E2F6. Plasmid constructs expressing BAF155, E2F6, and BRG1 were contransfected into 293 cells. Immunoprecipitation experiments were carried out in lysis buffer with a monoclonal antibody recognizing BAF155. Immunoprecipitates were resolved on SDS PAGE and Western blotted for E2F6, BRG1 and BAF155. C) E2F6 binds to BAF180. Plasmids expressing HA-tagged E2F6 or Flag-tagged BAF180 were co-transfected into 293T cells. Cell lysates were immunoprecipitated with polyclonal antibodies recognizing E2F6 or BAF180. Immunoprecipitates were than resolved on SDS PAGE and Western blotted with monoclonal antibodies recognizing HA or Flag epitope tags.

### Chromatin immunoprecipitation assays

Chromatin immunoprecipitation assays were carried out as previously described [5], [6], [29], [30], [31]. Nuclear chromatin extracts were incubated with antibodies from Santa Cruz Biotechnology described above at 4°C overnight. Immunoprecipitates were collected on 25 μl Protein G agarose beads (Roche) for 1–2 h at 4°C. After thorough washing, immunoprecipitates were de-crosslinked and chromatin was recovered on Qiagen miniprep spin columns. Quantitiative PCR (qPCR) analyses were performed in real time using the ABI PRISM 7900 Sequence Detection System and SYBR Green Master Mix following the manufacturer's protocol. Relative occupancy values were calculated as described previously [31]. Final values reflecting promoter occupancy are reported as the percent of input DNA, immunoprecipitated from each antibody after adjusting to input levels and normalization to albumin levels. Primer sets for qPCR are available upon request.

**Figure 4:**
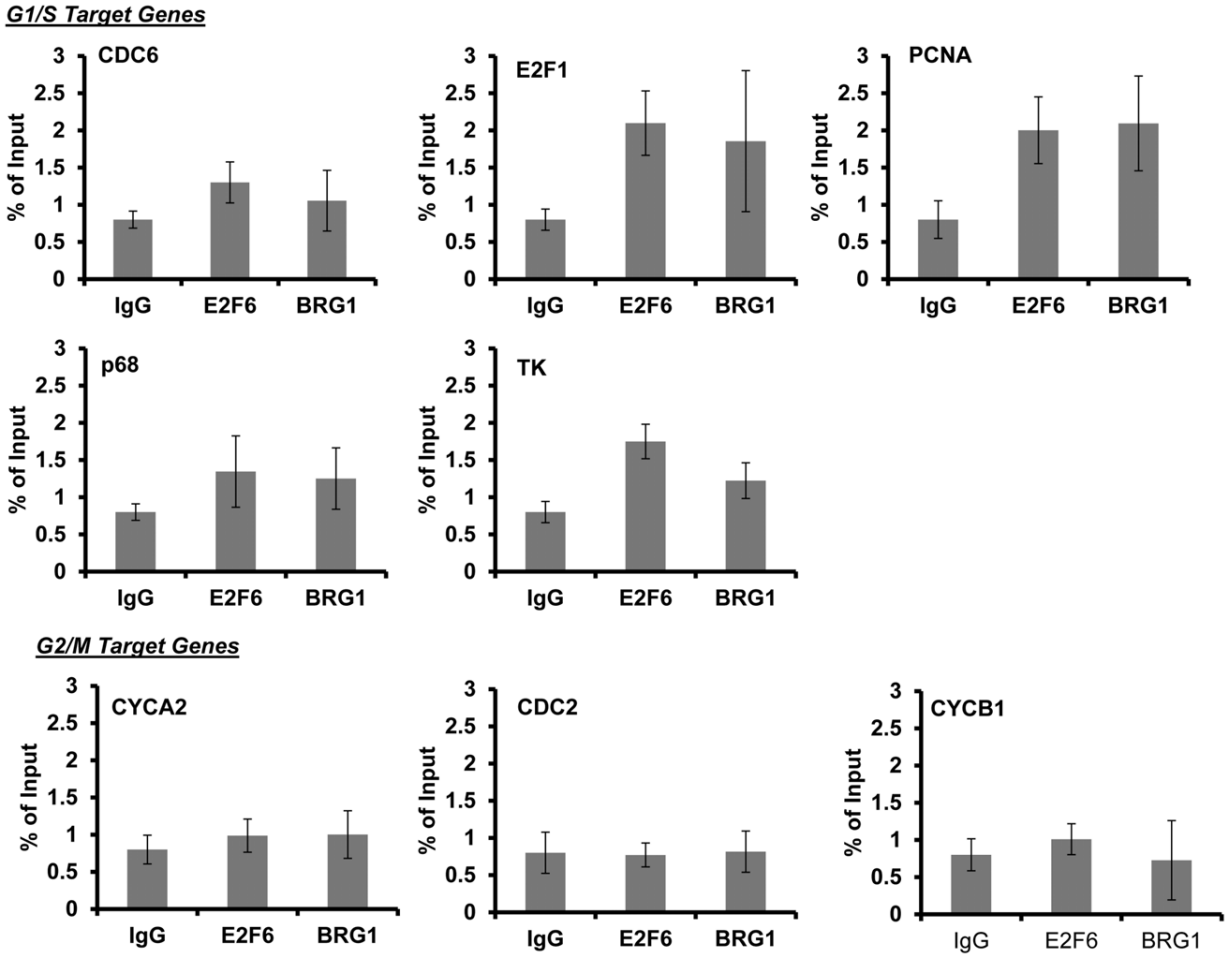
Chromatin immunoprecipitaion of E2F6 and BRG1 on G1/S and G2/M promoters. E2F6 and BRG1 bind to G1/S promoters but not G2/M promoters. Cells were synchronized by serum deprivation and then collected at 24 hours following serum stimulation. Chromatin immunoprecipitation was performed with antibodies to E2F6 and BRG1. A rabbit IgG was used as a negative control. Enrichment for G1/S and G2M promoters were assessed by quantitative PCR using SYBR Green.

### Luciferase Reporter Assays

Reporter assays to determine the effects of E2F6 on E2F1 promoter activity were carried out on 6-well dishes. Cells transfected 24 h after plating with 1 ug of the E2F1 reporter, 1 ug of a LacZ expressing plasmid and E2F6-pcDNA3 at concentrations of 0–90 ng per well. The experiment to determine the effects of a dominant BRG1 on E2F6-mediated repression was performed after transfection with 0.8 ug of a lac Z expressing plasmid, 0.8 ug of the E2F1 reporter, 250 ng of a plasmid expressing dominant negative BRG1 and 9 ng of E2F6. The total mass of transfected DNA in each well was kept constant by adding empty vector plasmid DNA, when necessary. All experiments were performed in triplicates, and mean ±s.d. values were determined. All transfections were performed overnight at 37°C and then cells were allowed to recover in 10% FBS–DMEM. At 18–36 h after transfection, cells were washed with 1 × PBS and then lysed with 1× Passive Lysis Buffer according to the manufacturer's protocol (Promega, Madison, WI, USA). Luciferase activity was determined from triplicate samples and expressed as the average relative luciferase units after normalization to β-galactosidase activity.

**Figure 5:**
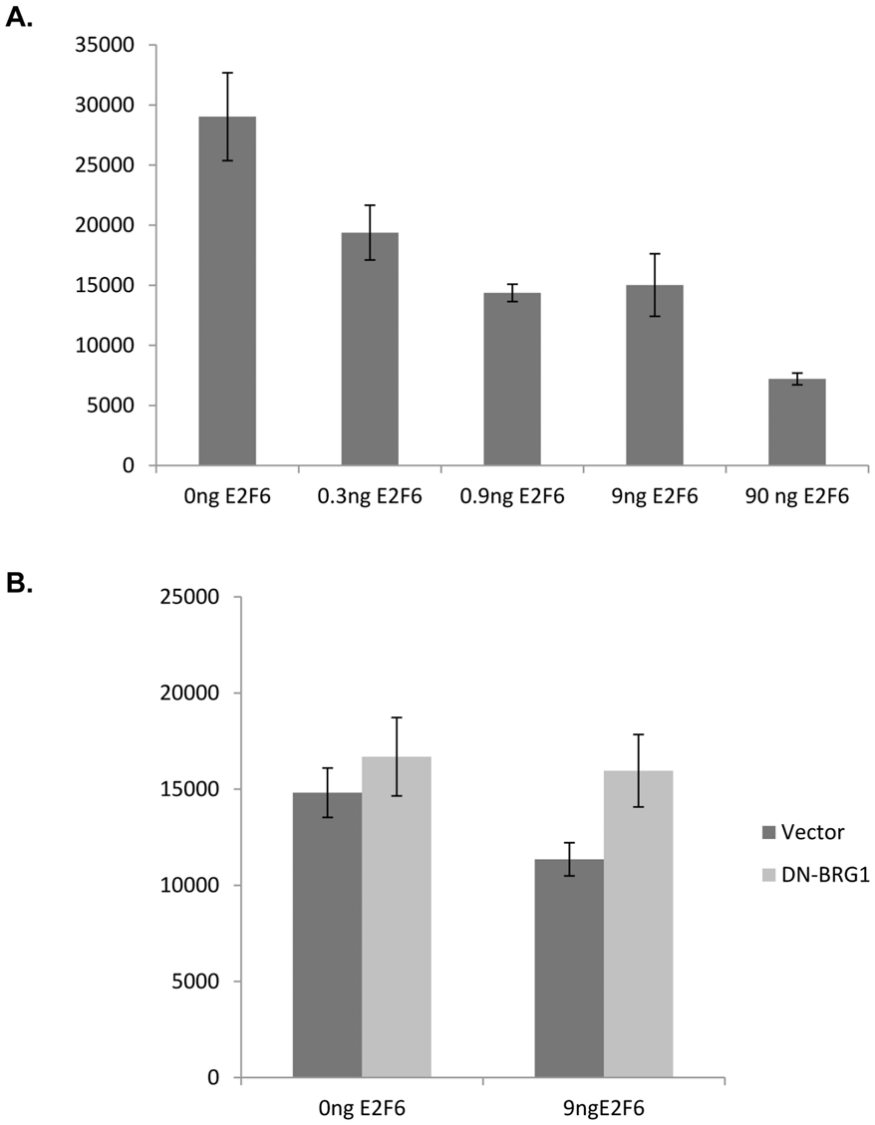
E2F6 repression of the E2F1 promoter is hindered by a dominant negative BRG1. Transfections were performed using T98G cells plated onto 6-well dishes. All wells were co-transfected with a construct expressing Lac Z and a E2F1 reporter plasmid in addition to the indicated plasmids. Luciferase activity was determined from triplicate samples and expressed as the average relative luciferase units after normalization to β-galactosidase activity. A) Increasing concentrations of E2F6 is correlated with a decrease in E2F1 reporter activity. A plasmid construct expressing E2F6 was transfected into T98G cells in 6 well dishes at the indicated amounts. The total mass of transfected DNA in each well was kept constant by adding empty vector plasmid DNA, when necessary. B) The ability of E2F6 to downregulate E2F1 promoter activity is inhibited by the presence of a dominant negative BRG1. A plasmid expressing a dominant negative BRG1 or an empty vector control was co-transfected with a plasmid construct expressing E2F6 into T98G cells. Luciferase activity was determined as described above.

## Results

### Identification of E2F6-interacting proteins

Prior work has shown that E2F6 functions as an active repressor of transcription [1], [19], [24], [32]. Unlike E2Fs 1–5, which function as repressors by recruiting histone deacetylases via their pocket protein binding partners, E2F6 is unable to bind pocket proteins and has been proposed to repress transcription through RB independent mechanisms. We carried out a yeast-two hybrid screen to identify novel E2F6-interacting proteins that may be recruited specifically by E2F6 to target promoters in transcriptional repression. Given that E2F6 was previously shown to be highly expressed in testis tissue, we screened a human testis cDNA library using a full-length E2F6 clone as bait [18]. From this screen, we identified 14 independent clones that represented previously annotated proteins with a potential to regulate gene transcription (Table 1). Among these 14 clones, three clones containing fragments representing EPC1, DP1 and DP2 were identified [1], [18], [19], [24]. Because these proteins have been shown to previously interact with E2Fs, this provided a strong validation of the screen. One additional clone contained a partial sequence coding for amino acids 462–578 of the BRG1 protein. Given that prior work has suggested a role for BRG1 in facilitating transcriptional regulation by a wide variety of proteins, we cloned full-length BRG1 and further confirmed its interaction with E2F6.

### E2F6 immunoprecipitates with BRG1

To determine an interaction between BRG1 and E2F6, we first incubated S^35^-labeled *in vitro* translated BRG1 with an E2F6-glutathionine S transferase (GST) fusion protein. Precipitation with GST beads revealed *in vitro* translated S^35^-labeled BRG1 associated with GST-E2F6 but not GST alone (Figure 1a). To confirm an interaction between E2F6 and BRG1 in cells, we coexpressed E2F6 and BRG1 in T98G cells. E2F6 was shown to immunoprecipitate with BRG1 when an antibody recognizing BRG1 was used (Figure 1b). Given that we saw an interaction between BRG1, we also tested the ability of endogenous E2F6 and BRG1 proteins to interact under normal physiological conditions in another cell line. In agreement with our studies above, an antibody recognizing endogenous E2F6 was able to immunoprecipitate BRG1 in 293 cells (Figure 1C).

To confirm the interaction between BRG1 and E2F6 was not resulting from a cross reaction between the antibodies recognizing E2F6 and BRG1, we determined an interaction between epitope tagged E2F6 and BRG1 proteins using antibodies to HA and Flag. An antibody recognizing HA tagged-E2F6 was able to immunoprecipitate Flag tagged-BRG1 only in cells that coexpressed HA-E2F6 and Flag-Brg1 and not in cells expressing either protein alone (Figure 1D). We quantitated the blots by densitometry to obtain an approximation of the fraction of proteins bound (Table S1).

### BRG1 specifically interacts with E2F4 and E2F6

Numerous studies have shown that despite overlapping functions observed among the E2F proteins, individual E2Fs also possess unique transcriptional regulatory functions. Specificity of function is dictated in part by the specificity in the proteins recruited by individual E2Fs. E2F3, for example, was previously observed to bind TFE3 to dictate specific binding of E2F3 to the DNA polymerase alpha p68 promoter [11]. E2F1 specifically bound to Jab1 and this interaction was found to be important for E2F1's role in apoptosis [33]. To determine if BRG1 binds specifically to E2F6, we determined the ability of HA-tagged E2F1, 2, 3, and 4 to interact with BRG1. As shown in Figure 2, only HA-tagged E2F4 and HA-tagged E2F6 were able to associate with BRG1. Although E2F4 did not interact with BRG1 in yeast (Table 1), we assume that the interaction between BRG1 with E2F4 may be weaker and therefore not detected unless ectopically expressed in cells.

### BRG1 functions in a complex with other proteins

Prior work has shown that members of the E2F family, including E2F6, dimerize with DP proteins for efficient DNA binding activity [18], [19], [24]. To determine if BRG1 is in a complex with E2F6/DP or whether it may be functioning in transcriptional regulation independent of DP, we assessed an association between DP and BRG1. An antibody to DP1 was able to immunoprecipitate both BRG1 and E2F6 (Figure 3A).

BRG1 is a component of the SWI/SNF complexes. In the mammalian system, SWI/SNF complexes contain, in addition to BRM or BRG1, as many as 8–10 subunits referred to as BRM- or BRG1- associated factors or BAFs [34], [35]. To determine if E2F6 is also associated with any BAFs via its interaction with BRG1, or whether its interaction with BRG1 is independent of the SWI/SNF complex, we determined E2F6's interaction with BAF 155 and BAF180. As shown in Figure 3B and 3C, immunoprecipitation with an antibody to BAF155 and BAF180 showed an association of these BAFs with E2F6. Our results suggest that E2F6 is a component of the polybromo-containing SWI/SNF complex, PBAF [36].

### E2F6 and BRG1 associates with the E2F1 promoter

Our prior work has shown that E2F6 specifically recognizes promoters of E2F target genes that are activated at G1/S of the cell cycle. This interaction during S phase is coincident with a decline in expression of the genes [5]. To test the possibility that BRG1 may interact with E2F6 on these promoters, we performed a chromatin immunoprecipitation assay to assess the presence of Brg1 on the promoters of genes previously found to be activated during G1-S phase of the cell cycle. Cells were synchronized by serum deprivation followed by induction with serum for 24 hours whereby cells are in S phase of the cell cycle. As shown in Figure 4, BRG1 associates with the G1/S promoters concurrently with E2F6 during S phase of the cell cycle. BRG1, however, was not observed on the CYCA2, CDC2 and CYCB1 promoters (G2/M promoters), where E2F6 does not bind.

### E2F6 repression of the E2F1 promoter is blocked by a dominant negative BRG1

To understand the potential function of the E2F6 and BRG1 complex, we used a luciferase reporter construct that consists of the E2F1 promoter [37]. Consistent with a role for E2F6 as a transcriptional repressor, we observed repression of the E2F1 reporter upon ectopic expression of E2F6 (Figure 5A). Furthermore, increasing concentrations of ectopically expressed E2F6 directly correlated with increases in the extent the E2F1 reporter was repressed (Figure 5A).

BRG1 functions as the ATPase subunit of the SWI/SNF complex in chromatin remodeling and mutations in the ATPase domain (amino acid 785) renders BRG1 a dominant negative. We confirmed expression of a flag-tagged dominant negative BRG1 (DN-BRG1) by Western blot and observed that ectopic expression of DN^-^BRG1 was able to interfere with E2F6 mediated repression of the E2F1 promoter (Figure 5B; Figure S1). Our results suggest that BRG1 plays a role in facilitating E2F6 mediated transcriptional repression of the E2F1 gene and that E2F6 is less efficient in transcriptional repression in the presence of a dominant negative BRG1.

## Discussion

The E2F transcription factor family has been shown to be comprised of three distinct subfamilies including the activators (E2F1-3), the Rb-dependent repressors (E2F4, E2F5), and the Rb-independent repressors (E2F6-8). Unlike the other E2Fs, relatively little is known regarding the mechanisms by which the Rb-independent E2Fs carry out transcriptional repression. Although previous studies have clearly delineated a role for E2F6 in developmental processes [25], [38], studies focusing on the role of E2F6 in regulating the cell cycle have yielded mixed results. For example, E2F6 has been shown to form complexes with Mga and Max in cells in G_0_, suggesting that E2F6 might repress Myc- and Brachyury-responsive genes, as well as E2F-responsive genes, in quiescent cells [39]. Other studies show that overexpression of E2F6 in quiescent NIH3T3 cells blocked their ability to re-enter S phase [19]. On the other hand, different studies have shown that overexpression of E2F6 in U2OS cells leads to an accumulation of cells in S phase [18]. It has been speculated that variations in the cell types and conditions used for study, along with the complexity inherent in cell cycle regulation, may contribute to differing results between some of the studies published to date. We previously observed that E2F6 regulates G1/S gene promoters during S phase in T98G cells, consistent with a role for E2F6 in S phase [5]. To define the mechanisms underlying E2F6 function in this context, we performed a yeast two-hybrid assay using full length E2F6 as bait and identified BRG1. Our results suggest that E2F6 may recruit BRG1 and other BAFs to modify chromatin structure in transcriptional regulation of the E2F1 promoter during cell cycle progression.

In mammalian cells, the SWI/SNF complex contains BRG1, or a closely related protein called Brahma (BRM), along with 8–10 other subunits referred to as BRM- or BRG1-associated factors or BAFs which modulate the targeting and activity of the ATPase subunit [34], [35]. The SWI/SNF complex has been shown to play essential roles in a variety of processes including differentiation, proliferation and DNA repair via the ability to interact with a number of proteins. BRG1 has been shown to function in these biological processes as either a transcriptional activator or repressor depending on its partner proteins and the context under study. It has been speculated that the duality of BRG1 function is attributed to its role as a neutral co-regulator capable of synergizing with other transcriptional regulators in its vicinity. Work from Nagl et al., for example, have shown that a choice in association between two different variants of a major subunit of the ARID protein family determines whether the SWI/SNF complex forms further associations with activator or repressor complexes [40]. Our observation that E2F6 binds to BRG1 suggests a role for BRG1 in transcriptional repression in this context. We observed BRG1 also interacts with E2F4, another transcriptional repressor in the E2F family of proteins, when ectopically expressed in cells, although this was not observed in yeast. We speculate that the interaction between E2F4 and BRG1 may be weak and may not occur under normal physiological conditions. The observation that BRG1 is capable of binding E2F4 when overexpressed, however, is consistent with our previous observations indicating E2F4 can compensate for E2F6 in E2f6-null cells [5].

A number of studies have implicated a role for BRG1 in E2F regulation via its interaction with other proteins, although a direct interaction between E2Fs and BRG1 has not been documented. EVI1, a DNA-binding protein that belong to the Kruppel family of proteins, interacts with BRG1 to block BRG1's repressive regulation on E2F1 activity [41]. Prohibitin, a potential tumor suppressor gene, recruits BRG1 for repression of E2F responsive promoters by estrogen antagonists [42], [43]. TopBP1, a DNA topoisomerase IIβ binding protein, represses E2F1 transcription by a BRG1 dependent mechanism. Our observation that E2F6 can coimmunoprecipitate with BRG1 and its subunits, BAF155 and BAF180, suggests E2F6 can be a component of the polybromo-containing SWI/SNF complex known as PBAF under specific biological contexts. This is particularly interesting given a recently documented role for PBRM1 loss in renal and breast cancers [44], [45]. Our results presented here highlight diverse roles in normal homeostasis for another E2F family member that can be dictated by their interacting proteins.

## Supporting Information

Figure S1 **Western blot confirms expression of a dominant negative BRG1 from flag-tagged DN^-^BRG1. pcDNA3 used in** **Figure 5**. DN^-^BRG1. pcDNA3 was tranfected into 293T cells. Lysates were collected 48 hours post transfection and resolved on SDS PAGE. Western blot was carried out using an antibody recognizing the flag epitope tag.
(TIF)

Table S1 The density of bands on protein gels from Figure 1 were assessed. The density values were used to calculate the percentage of E2F6 bound to the total amount BRG1 immunoprecipitated and vice versa.
(TIF)
