# Supplementary figures and images for: E2F6 Associates with BRG1 in Transcriptional Regulation
Source: PLoS One. 2012 Oct 17;7(10):e47967. doi: 10.1371/journal.pone.0047967 (PMC3474740; doi:10.1371/journal.pone.0047967)

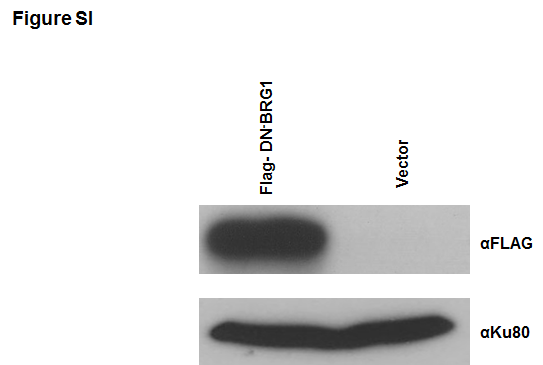

Supplement: Figure S1 — Western blot confirms expression of a dominant negative BRG1 from flag-tagged DN-BRG1. pcDNA3 used in Figure 5 . DN-BRG1. pcDNA3 was tranfected into 293T cells. Lysates were collected 48 hours post transfection and resolved on SDS PAGE. Western blot was carried out using an antibody recognizing the flag epitope tag. (TIF) [file pone.0047967.s001.tif]

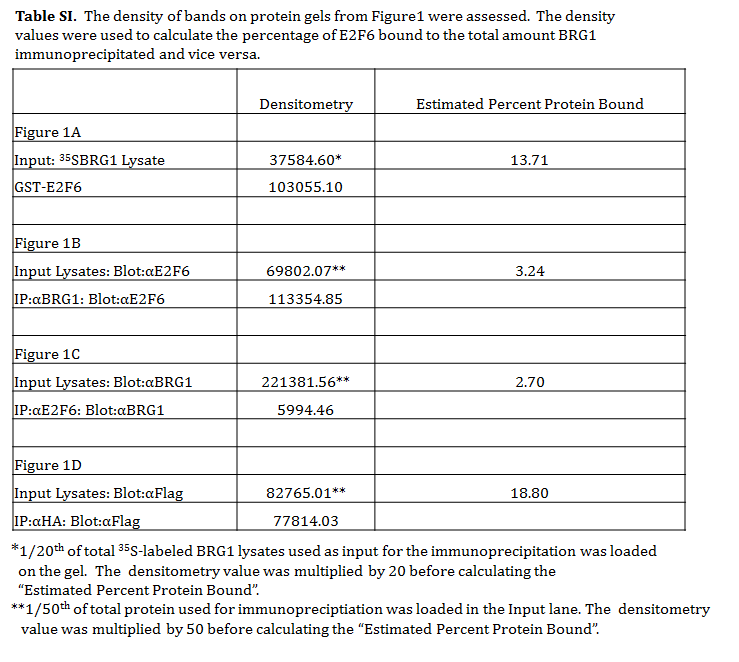

Supplement: Table S1 — The density of bands on protein gels from Figure 1 were assessed. The density values were used to calculate the percentage of E2F6 bound to the total amount BRG1 immunoprecipitated and vice versa. (TIF) [file pone.0047967.s002.tif]
